# Synergy of arbuscular mycorrhizal symbiosis and exogenous Ca^2+^ benefits peanut (*Arachis hypogaea* L.) growth through the shared hormone and flavonoid pathway

**DOI:** 10.1038/s41598-019-52630-7

**Published:** 2019-11-07

**Authors:** Li Cui, Feng Guo, Jialei Zhang, Sha Yang, JingJing Meng, Yun Geng, Xinguo Li, Shubo Wan

**Affiliations:** 1 Biotechnology Research Center, Shandong Academy of Agricultural Sciences, Jinan, 250100 China; 2 Scientific Observing and Experimental Station of Crop Cultivation in East China, Ministry of Agriculture, Jinan, 250100 China; 3 College of Life Sciences, Shandong Normal University, Jinan, 250014 China; 4 Shandong Academy of Agricultural Sciences and Key Laboratory of Crop Genetic Improvement and Ecological Physiology of Shandong Province, Jinan, 250100 China

**Keywords:** Gibberellins, Plant molecular biology, Plant signalling, Arbuscular mycorrhiza

## Abstract

Peanut yield is severely affected by exchangeable calcium ion (Ca^2+^) deficiency in the soil. Arbuscular mycorrhizal (AM) symbiosis increases the absorption of Ca^2+^ for host plants. Here, we analyzed the physiological and transcriptional changes in the roots of *Arachis hypogaea* L. colonized by *Funneliformis* *mosseae* under Ca^2+^-deficient and -sufficient conditions. The results showed that exogenous Ca^2+^ application increased arbuscular mycorrhizal fungi (AMF) colonization, plant dry weight, and Ca content of AM plants. Simultaneously, transcriptome analysis showed that Ca^2+^ application further induced 74.5% of differentially expressed gene transcripts in roots of AM peanut seedlings. These genes are involved in AM symbiosis development, hormone biosynthesis and signal transduction, and carotenoid and flavonoid biosynthesis. The transcripts of AM-specific marker genes in AM plants with Ca^2+^ deprivation were further up-regulated by Ca^2+^ application. Gibberellic acid (GA_3_) and flavonoid contents were higher in roots of AM- and Ca^2+^-treated plants, but salicylic acid (SA) and carotenoid contents specifically increased in roots of the AM plants. Thus, these results suggest that the synergy of AM symbiosis and Ca^2+^ improves plant growth due to the shared GA- and flavonoid-mediated pathway, whereas SA and carotenoid biosynthesis in peanut roots are specific to AM symbiosis.

## Introduction

Peanut (*Arachis hypogaea* L.) is an important oil crop and protein source for humans that contributes 20% to oil production and 11% of the human protein supply per year. The yield is often limited by exchangeable Ca^2+^ deficiency in soil, which causes early embryo abortion in peanut^1,2^. Therefore, Ca^2+^ plays a crucial role in the growth and development of peanut. Calcium is an important macronutrient required for plant growth and development and represents 0.1 to 5% of all plant dry biomass^3^. Additionally, as a second messenger, Ca^2+^ has been shown to mediate various aspects of cell and plant development, such as cell division, cell polarity, cell elongation, photomorphogenesis, and biotic and abiotic stress responses^4–6^.

In peanut, Ca^2+^ partly regulates turnover of the PSII reaction center components to reduce the stress of photoinhibition to PSII^7^, and is involved in hormone-induced peanut pod formation by increasing gibberellic acid (GA) and auxin contents^1^. However, Ca^2+^ is largely confined to uptake via the young root tips and can only be taken up by the young root system from the soil and delivered to the shoot via the xylem, and it is not remobilized from old to young tissues^8^. Thus, Ca^2+^ deficiency commonly affects plant growth and development if the soil cannot be supplemented with exogenous Ca^2+^. Fortunately, most plants have coped with limited Ca^2+^ availability via the establishment of symbiotic associations with microbes, more specifically known as AM association. The fungi forming AM symbiosis belong to the subphylum Glomeromycotina^9^.

This symbiosis plays a significant role in the uptake of nutrients and the carbon cycle, and consequently impacts ecosystem sustainability^10^. To establish the symbiosis, plant roots recognize chemical signals from AMF, e.g. lipochitooligosaccharides and chitooligosaccharides, which trigger coordinated differentiation and form the symbiotic state^11^. In turn, AMF require signal communication from the plants that produce strigolactones (which are derived from the carotenoid synthesis pathway), flavonoids, and other diffusible signals exuded by plant roots that induce the germination of AMF spores and branched fungal hyphae^12^. Then, the AM symbiosis is established by the common symbiosis signaling pathway induced by calcium oscillation after perception of diffusible signals from the symbionts^13^. In the process of establishing the AM symbiosis, many AM-specific marker genes must be initiated by Ca^2+^ concentration change^14^, such as *RAM1* (*REDUCED ARBUSCULAR MYCORRHIZA 1*), *RAM2* (*glycerol-3-phosphate acetyltransferase*), *CCD1* (*carotenoid cleavage dioxygenase*), *PT1* (*phosphate transporter*), and *DELLA*^15–17^. These findings suggest that Ca^2+^ plays an important role in AM development.

From recognition of the fungi to establishment of the symbiosis, complicated transcriptional reprogramming occurs in plant roots, and many specifically expressed genes involved in development of the symbiosis have been identified in legumes^18–20^. Some of the changes associated with plant hormones were considered to play important roles in this symbiosis^21^, such as auxin, cytokinins (CKs), gibberellins (GAs), and strigolactones, and were also altered in the roots of AM plants^16,22^. In addition, increases in flavonoid and anthocyanin were considered to be indispensable in regulating the establishment of AM symbiosis^22,23^.

Even though the molecular basis of the improvement of plant nutrient acquisitions have been well characterized for phosphorus, nitrogen, sulfur, and potassium^18,24,25^, the role of plant uptake of Ca^2+^ needs further study. Several reports showed that a moderate level of Ca^2+^ supply enhanced the colonization of AMF^26,27^, and Ca^2+^ benefited the maintenance of a functioning mycorrhiza^28^. However, the transcriptional changes in plant roots colonized by an AMF accompanied with sufficient Ca^2+^ are still unknown.

Cui *et al*. (2019) demonstrated that AM symbiosis increased the Ca^2+^ content in peanut seedlings, and Ca^2+^ application can also promote the development of AM symbiosis^29^. However, the molecular mechanism of how AMF and Ca^2+^ application synergistically promote the growth of peanut seedlings is unclear. In this study, we investigated a combination of transcriptional changes, hormone and metabolomic analyses in roots of peanut seedlings inoculated by AMF and Ca^2+^ application and compared the observed changes with those in AM plants or Ca^2+^-treated plants. We observed that changes in secondary metabolites in roots of AM and Ca^2+^-treated plants coincide with the transcriptional regulation of related biosynthesis pathways. These alternations, such as the increases in GA_3_ and flavonoid content, were considered to be involved in the growth enhancement of peanut seedlings by the synergy of AMF with Ca^2+^ application.

## Results

### AM symbiosis improves the dry biomass of peanut

**Figure 1:**
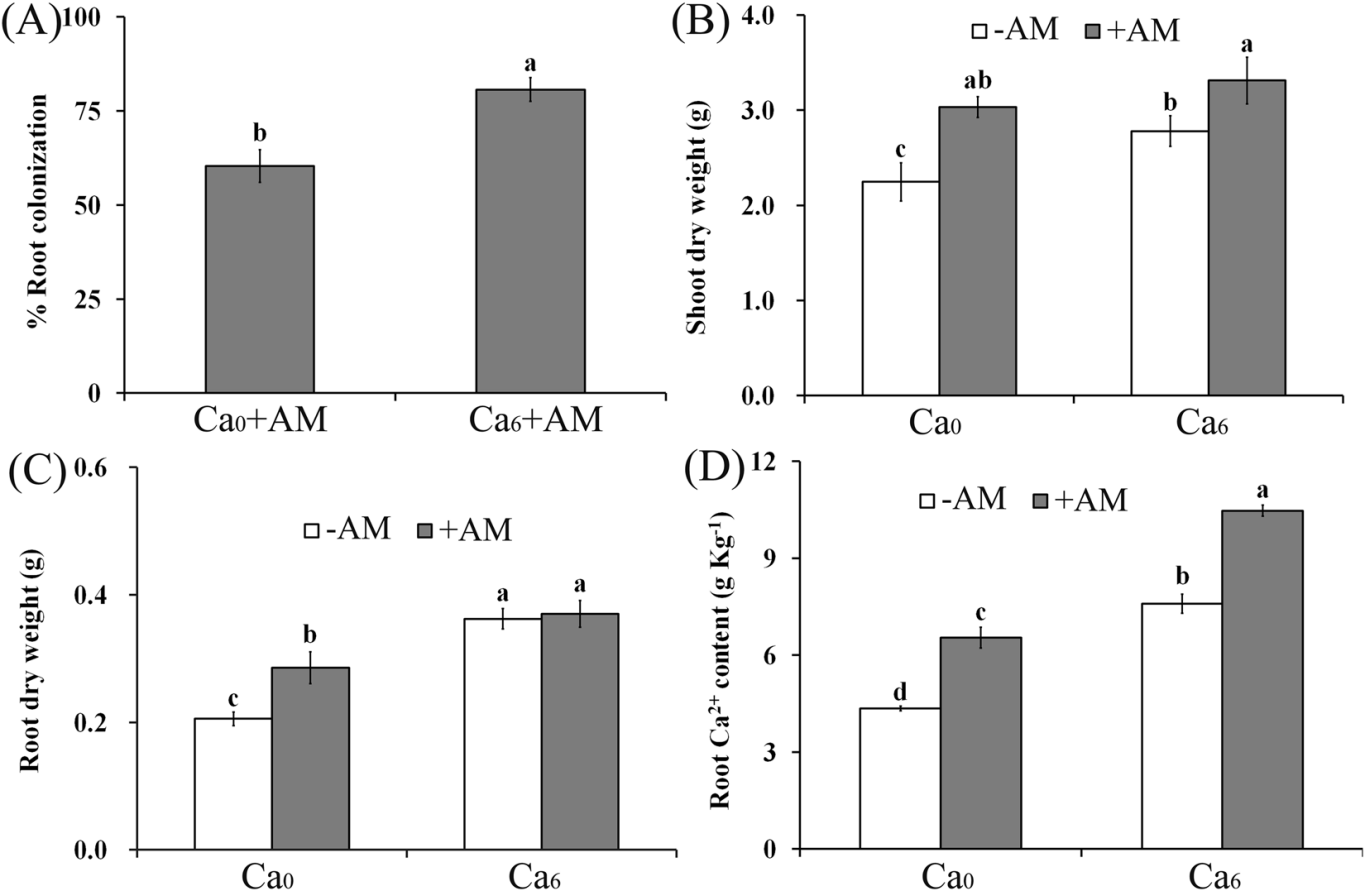
Impact of AM symbiosis on plant growth under Ca^2+^ deficient and sufficient conditions. (**A)** The rate of AMF colonization in peanut roots was assayed under Ca^2+^ deprivation and sufficiency. Shoot (**B**) and root (**C**) dry weight were determined in AM and NM plants under different Ca^2+^ treatments. (**D**) Ca^2+^ content was measured in roots of AM and NM plants under Ca^2+^ deficient and Ca^2+^ sufficient conditions. Letters represent significant differences between treatments and the control (one-way ANOVA, *P* < 0.05). Bars indicate means ± SD from six plants. DW, dry weight.

The quantification of AMF colonization showed that 60.33% and 80.67% of plant roots were inoculated by *F*. *mosseae* under both Ca^2+^-deficient and Ca^2+^-sufficient conditions, respectively (Fig. 1A), indicating that Ca^2+^ application could significantly improve the number of fungal colonizers.

Shoot dry weight significantly increased in the AM plants compared with the nonmycorrhized (NM) plants and Ca^2+^ application further increased the shoot dry weight (Fig. 1B). Moreover, root dry weight was significantly increased in AM plants under Ca_0_ conditions and Ca^2+^ further improved the root dry weight; AM symbiosis did not increase the root dry weight (Fig. 1C). Additionally, the Ca^2+^ content was significantly higher in Ca^2+^-sufficient seedlings compared with Ca^2+^-deficient ones, and AM association improved Ca^2+^ level in roots (Fig. 1D).

### Comparative analysis of differentially expressed genes (DEGs) in the AM and Ca^2+^-treated plants

**Figure 2:**
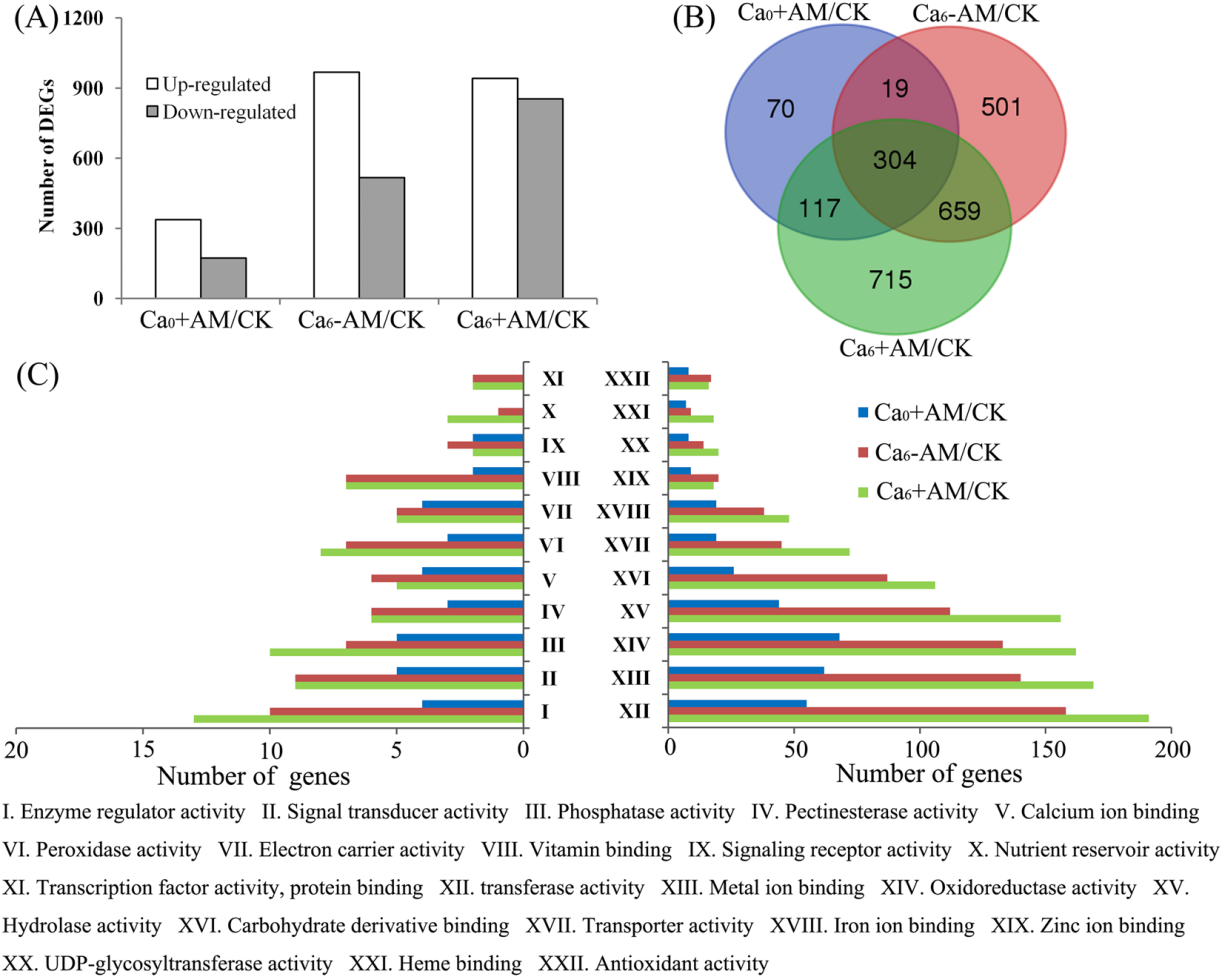
Transcriptional profiling of peanut roots with or without colonization by AMF under Ca^2+^ deficient and sufficient conditions. (**A**) The number of DEGs up-regulated and down-regulated in roots of Ca_0_ − AM, Ca_6_ − AM, and Ca_6_ + AM plants compared with Ca_0_-AM plants (the control). (**B**) Venn diagram showing the number of DEGs shared and specifically up- or down-regulated in roots of Ca_0_ − AM, Ca_6_ − AM, and Ca_6_ + AM plants. (**C**) Significantly enriched GO molecular function terms for the number of DEGs analyzed in different treatments.

Using Ca_0_-AM as the control, there were 510, 1483, and 1795 significantly differentially expressed genes (DEGs) from roots of the Ca_0_ + AM, Ca_6_ − AM, and Ca_6_ + AM plants, respectively (Fig. 2A). In all, 304 DEGs were shared by Ca_0_ + AM, Ca_6_ − AM, and Ca_6_ + AM plants and the number of DEGs gradually increased in the plants (Fig. 2B), indicating that AM symbiosis combined with exogenous Ca^2+^ induced more transcriptional changes. In total, 421 DEGs were shared by Ca_0_ + AM and Ca_6_ + AM treatments, representing 82.55% and 23.45% of total DEGs in Ca_0_ + AM plants (510) and Ca_6_ + AM plants (1795), respectively. The expression levels of 380 DEGs in Ca_0_ + AM plants could be further regulated by Ca^2+^ application (Supplementary Fig. S1); only 40 DEGs were conversely regulated (Supplementary Table S1). This result implied that Ca^2+^ application could further strengthen the effects of AM on plant growth. In addition, 22 categories involved in molecular functions of GO enrichment analyses were identified, and the number of DEGs involved in transferase activity was the highest, followed by metal ion binding and oxidoreductase activity. Four categories, including calcium ion binding, signaling receptor activity, zinc ion binding, and antioxidant activity were the highest in Ca_6_ − AM plants; among the other 18 categories, the number of DEGs involved in each molecular function of GO was the highest in the Ca_6_ + AM plants, followed by the Ca_6_ − AM plants, and the Ca_0_ + AM plants (Fig. 2C).

**Figure 3:**
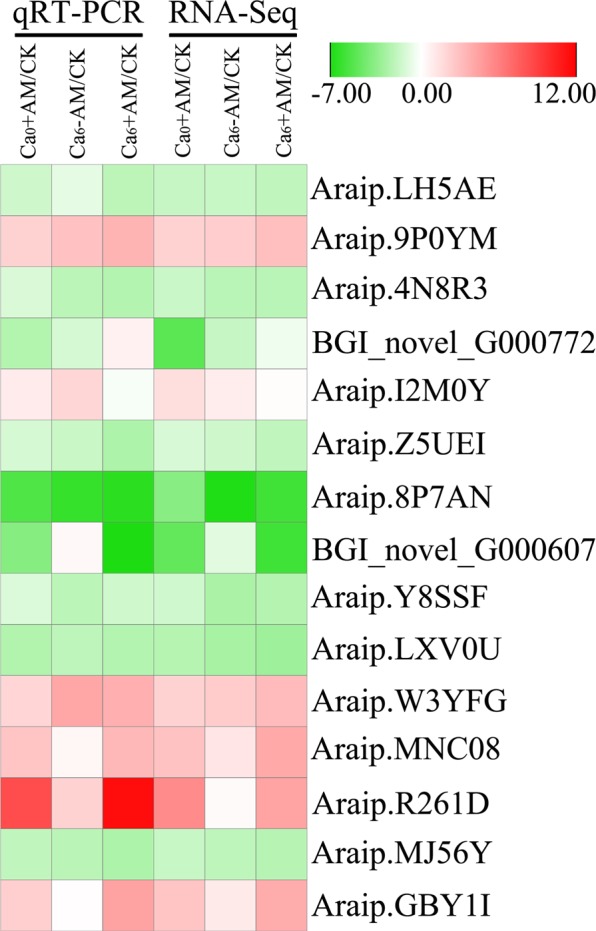
qRT-PCR verification of selected genes. Comparison of gene expression level from transcriptome analyses and qRT-PCR experiments.

To confirm RNA-Seq results, 15 genes were selected randomly from various functional categories and qRT-PCR analysis was conducted using RNA samples from the RNA-Seq experiments. The results were consistent with the expression levels of genes from RNA-Seq data (Fig. 3).

### Effects of Ca^2+^ combined with AM symbiosis on the transcripts of AM-specific markers and Ca-related genes

We analyzed the roles of Ca^2+^ application on the establishment of AM symbiosis. In total, 25 AM-specific marker genes were identified in Ca_6_ + AM plants, with 12 GRAS family transcription factors (TFs) ten of which were up-regulated and two down-regulated. However, only 12 AM-specific marker genes were induced in Ca_0_ + AM plants and the transcripts of ten up-regulated genes were further increased by Ca^2+^ application (Table 1), including *MYB*, *AP2*, *CCD*, *DELLA1*, *RAM1*, *RAM2*, and *DELLA*. In addition, some AM-specific marker genes were specifically expressed in AM plants by Ca^2+^ application, e.g. *DXS2*, *DIM2*, *SbtM1*, and *PUB1*.

**Table 1:** Differentially expressed genes of AM-specific markers in roots of Ca_0_ + AM and Ca_6_ + AM treated plants compared with controls.

Gene Name	Gene ID	Annotation	Ca_0_ + AM/CK	Ca_6_ + AM/CK
DXS2	Araip.581AC	1-Deoxy-D-xylulose-5-phosphate synthase	—	**−1**.**37**
DIM2	Araip.7E8G5	receptor-like kinase	—	**4**.**14**
SbtM1	Araip.2Y3EX	subtilisin-like protease	—	**2**.**23**
IPD3	Araip.02MA2	cyclops protein	—	**1**.**51**
PUB1	Araip.658mf	E3 ubiquitin ligase	—	**2**.**24**
MYB	Araip.62YF9	MYB transcription factor	**2**.**87**	**4**.**39**
AP2	BGI_novel_G002001	AP2 transcription factor	**1**.**93**	**2**.**98**
CCD1	Araip.S2QC7	carotenoid cleavage dioxygenase	**2**.**99**	**5**.**63**
CCD7	Araip.RJ87T	carotenoid cleavage dioxygenase 7	**1**.**13**	**2**.**65**
CCD8	Araip.MNC08	carotenoid cleavage dioxygenase 8	**2**.**86**	**4**.**03**
PT1	Araip.QVW26	phosphate transporter	—	**4**.**23**
PT4	Araip.WR1Z1	inorganic phosphate transporter	**5**.**33**	**5**.**22**
RAM2	Araip.1QC5L	glycerol-3-phosphate acyltransferase	**3**.**43**	**5**.**32**
RAM1	Araip.N9QES	GRAS family transcription factor	**4**.**15**	**6**.**30**
DELLA1	BGI_novel_G000145	GRAS family transcription factor	**2**.**89**	**5**.**05**
DELLA	Araip.LT9MF	GRAS family transcription factor	**1**.**80**	**2**.**47**
DELLA	BGI_novel_G000391	GRAS family transcription factor	**1**.**81**	**2**.**84**
DELLA	Araip.DNQ5K	GRAS family transcription factor	**−2**.**49**	**−3**.**62**
DELLA	Araip.RWP2N	GRAS family transcription factor	—	**4**.**47**
DELLA	Araip.TD6FV	GRAS family transcription factor	—	**3**.**40**
DELLA	BGI_novel_G001778	GRAS family transcription factor	—	**1**.**15**
DELLA	Araip.W23GC	GRAS family transcription factor	—	**−1**.**51**
DELLA	Araip.KB0T7	GRAS family transcription factor	—	**1**.**46**
DELLA	Araip.KK7TK	GRAS family transcription factor	—	**1**.**18**
DELLA	BGI_novel_G001435	GRAS family transcription factor	—	**1**.**08**

Values represent significant changes in roots of AM plants under Ca^2+^ deficient and sufficient conditions compared with the control (NM-Ca). Positive and negative ratios indicate up- and down-regulated genes. − Represents no significant alterations at log_2_FoldChange ≥ 1 and *P* value ≤ 0.05 level.

We further investigated the effects of AM symbiosis on Ca and Ca^2+^ signal-related genes. The number of DEGs involved in Ca signals in the Ca_6_ − AM and Ca_6_ + AM plants was 29 and 32, respectively (Supplementary Table S2). However, there were 14 DEGs shared by Ca_6_ − AM and Ca_6_ + AM plants and the transcript levels of nine of these DEGs were further regulated by AM symbiosis. Additionally, AM symbiosis specifically up-regulated the transcripts of *Araip*.*IZ5U3* and *Araip*.*R6YEY* genes, which code the potassium channel KAT3 and AKT2/3, respectively. These results suggest that the Ca^2+^ signal pathway induced by exogenous Ca^2+^ is partially different from AM symbiosis.

### Effects of AM symbiosis and Ca^2+^ on genes involved in hormone biosynthesis

DEGs involved in plant hormone biosynthesis were screened, including auxin, CKs, GA, and SA (Table 2). One gene encoding auxin responsive protein indoleacetic acid (IAA) was specifically up-regulated in AM plants without Ca^2+^ application. Two genes belonging to the auxin responsive GH3 family were down-regulated in AM plants, and Ca^2+^ application further down-regulated its transcripts. The genes encoding cytokinin dehydrogenase, which catalyze the irreversible degradation of CK, were either up- or down-regulated. In addition, we observed an increase in transcripts of genes involved in the biosynthesis of GA. Compared with the control, all DEGs encoding gibberellin 20-oxidase were up-regulated in AM plants, and more transcripts were observed in Ca_6_ + AM plants. Two selected DEGs, namely, gibberellins 2-oxidase and gibberellin receptor GID1, were only up-regulated in Ca_6_ + AM plants. Meanwhile, one  TF TGA (Araip.FKG2G) involved in the biosynthesis of SA was specifically up-regulated by Ca^2+^ application, and was further up-regulated by AM symbiosis.

**Table 2:** List of selected altered genes involved in hormone signal transduction in roots of Ca_0_+AM, Ca_6_—AM, and Ca_6_+AMtreated plants compared with controls.

GeneID	Gene Description	Ca0 + AM/CK	Ca6 − AM/CK	Ca6 + AM/CK
**Auxin**				
Araip.I2M0Y	auxin responsive protein IAA	1.54	—	—
Araip.PP5S8	auxin responsive GH3 gene family	−2.20	−2.60	−3.42
Araip.V8NJN	auxin responsive GH3 gene family	−2.31	−5.41	−5.42
**Cytokinin**				
Araip.DKI8Z	cytokinin dehydrogenase	—	2.00	2.22
Araip.ZXC56	cytokinin dehydrogenase	−1.44	−1.97	−2.48
Araip.2I0VZ	histidine-containing phosphotransfer protein	—	−2.32	−3.17
Araip.W2KBF	cytokinin dehydrogenase	—	—	−2.05
**Gibberellin**				
Araip.9GU4E	gibberellin 20-oxidase	2.39	1.88	3.03
Araip.UXP0Y	gibberellin 20-oxidase	2.03	1.62	2.48
Araip.X2IEW	gibberellin 20-oxidase	1.84	1.39	2.73
Araip.B4LS2	gibberellin-regulated protein	—	1.85	2.07
Araip.HQ99N	gibberellin 20-oxidase	—	1.26	1.57
Araip.L4RII	gibberellin 20-oxidase	—	1.45	1.60
Araip.E8TE0	gibberellin 20-oxidase	—	—	4.62
Araip.4FI3B	gibberellin 20-oxidase	—	—	1.77
Araip.78FT4	gibberellin 20-oxidase	—	—	2.15
Araip.50IUR	gibberellin 20-oxidase	—	—	1.73
Araip.6PA6C	gibberellin 2-oxidase	—	—	1.87
Araip.99KY6	gibberellin receptor GID1	—	—	1.58
**Salicylic acid**				
Araip.FKG2G	transcription factor TGA	—	2.80	4.08

Values represent significant alterations in AM or Ca^2+^-treated plants compared with the control. Positive and negative ratios indicate up- and down-regulated genes. − Represents no significant alterations at log_2_FoldChange ≥ 1 and *P* ≤ 0.05 level.

**Figure 4:**
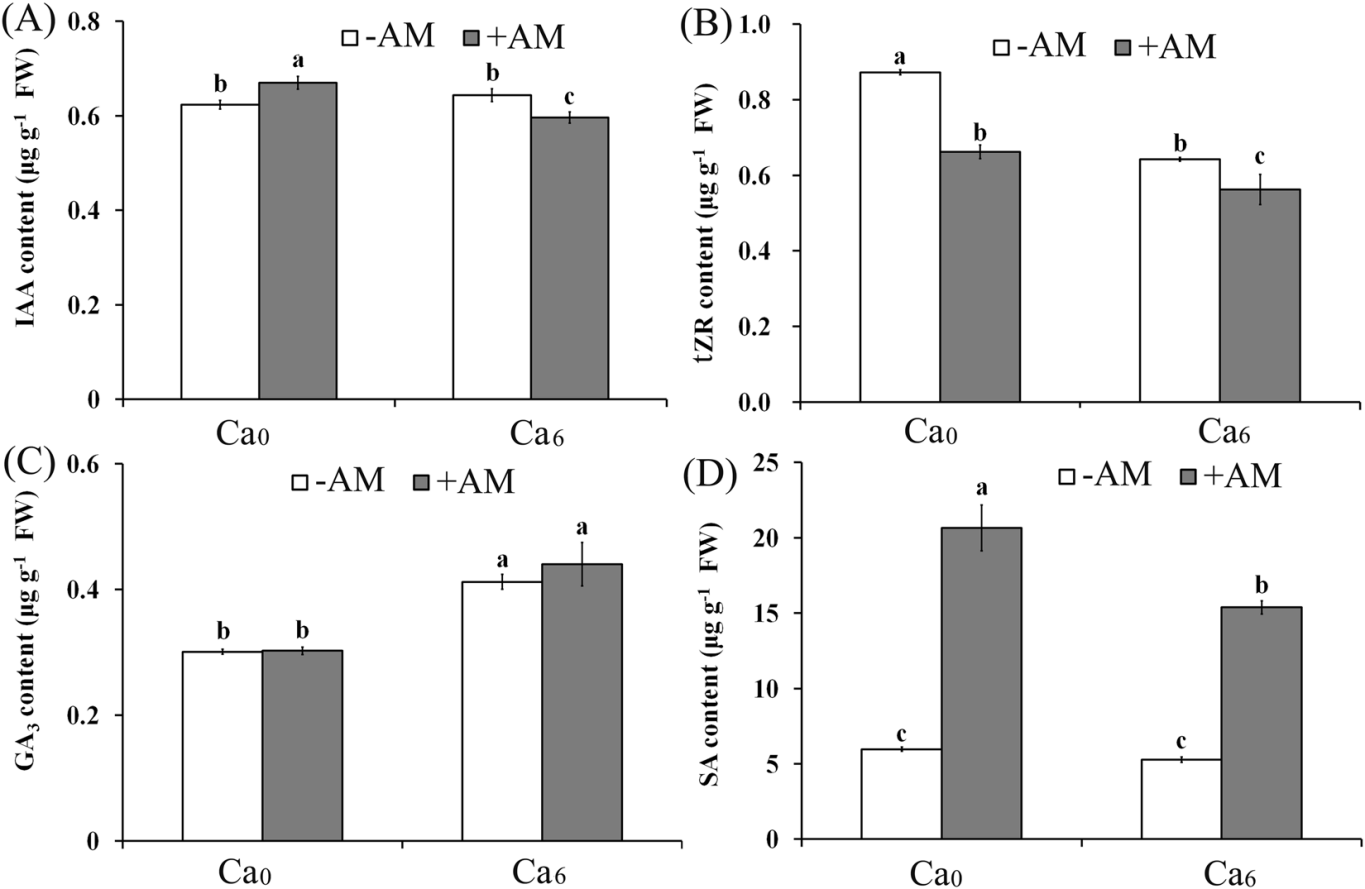
Determination of hormone levels in peanut roots. The IAA (**A**), tZR (**B**), GA_3_ (**C**), and SA (**D**) content were quantified in roots of the AM plants and NM plants under Ca^2+^ deficient and sufficient conditions. Bars indicate means ± SD from six plants. Letters represent significant differences between treatments and the control (one-way ANOVA, *P* < 0.05). FW: fresh weight.

In order to verify whether the hormone level is consistent with the transcriptional changes in DEGs, we tested the content of IAA, trans-zeatin riboside (tZR), GA_3_, and SA. IAA content significantly increased in AM plants with Ca_0_ treatment, but decreased in Ca_6_ treatment (Fig. 4A). Changes in tZR content were consistent with the transcript changes of cytokinin dehydrogenase genes: it significantly decreased in Ca_0_ + AM and Ca_6_ − AM plants, and further decreased in Ca_6_ + AM plants (Fig. 4B). Additionally, the GA_3_ content only significantly increased in Ca_6_ treatment (Fig. 4C), which was consistent with the transcriptional changes of GAs biosynthesis. SA content significantly increased only in the roots of AM plants (Fig. 4D).

### Effects of AM symbiosis and Ca^2+^ on genes involved in carotenoid and flavonoid biosynthesis

We found an increase in transcripts of DEGs involved in carotenoid biosynthesis. The genes encoding 3-oxoacyl-[acyl-carrier protein] reductase (BGI_novel_G000088), 15-cis-phytoene/all-trans-phytoene synthase (Araip.40 × 13), and 9-cis-beta-carotene 9′,10′-cleaving dioxygenase (*CCD7*, Araip.RJ87T) involved in carotenoid biosynthesis were only up-regulated in AM plants, and were further up-regulated by Ca_6_ treatment (Table 3). In addition, the genes encoding unknown protein (BGI_novel_G003217), capsanthin/capsorubin synthase (Araip.3B5FU), and beta-carotene isomerase (Araip.FA949, *DWARF27*) were specifically up-regulated in Ca_6_ + AM plants.

**Table 3:** Differentially expressed genes involved in carotenoid biosynthesis in roots of AMF and Ca^2+^ treated plants compared with controls.

Gene ID	Annotation	Ca_0_ + AM/CK	Ca_6_ + AM/CK	Ca_6_ − AM/CK
BGI_novel_G000088	3-oxoacyl-[acyl-carrier protein] reductase	2.49	3.80	—
Araip.Y8SSF	abscisate beta-glucosyltransferase	−1.45	−2.31	−2.58
BGI_novel_G001960	momilactone-A synthase	2.46	4.22	2.10
Araip.40X13	15-cis-phytoene/all-trans-phytoene synthase	2.23	5.38	—
Araip.MNC08	carotenoid cleavage dioxygenase 8	2.86	4.03	1.20
Araip.D2DUM	xanthoxin dehydrogenase	−1.83	−1.97	−2.15
Araip.RJ87T	9-cis-beta-carotene 9′,10′-cleaving dioxygenase	1.13	2.65	—
Araip.AB0RD	prolycopene isomerase	—	−1.46	−1.18
Araip.D5CVZ	momilactone-A synthase	—	—	−1.45
BGI_novel_G003217	unknown protein	—	2.96	—
Araip.3B5FU	capsanthin/capsorubin synthase	—	2.18	—
Araip.FA949	beta-carotene isomerase	—	2.79	—

Values represent significant alterations in AM or Ca^2+^-treated plants compared with the control. Positive and negative ratios indicate up- and down-regulated genes. − Represents no significant alterations at log_2_FoldChange ≥ 1 and *P* ≤ 0.05 level.

Transcriptional changes involved in flavonoid biosynthesis were also observed. The genes encoding chalcone synthase involved in early steps of flavonoid biosynthesis were all down-regulated in Ca_0_ and Ca_6_ treatments. In contrast, one gene (BGI_novel_G001027) encoding shikimate O-hydroxycinnamoyltransferase was up-regulated in the Ca_0_ and Ca_6_ treatments, and the expression level was the highest in AM plants treated with Ca_6_. However, the other gene encoding shikimate O-hydroxycinnamoyltransferase was specifically up-regulated in Ca_6_ + AM plants. In addition, the gene (Araip.6PA6C) encoding flavonol synthase responsible for the biosynthesis of flavanol, was specifically up-regulated in AM plants with Ca_6_ treatment (Supplementary Table S3).

**Figure 5:**
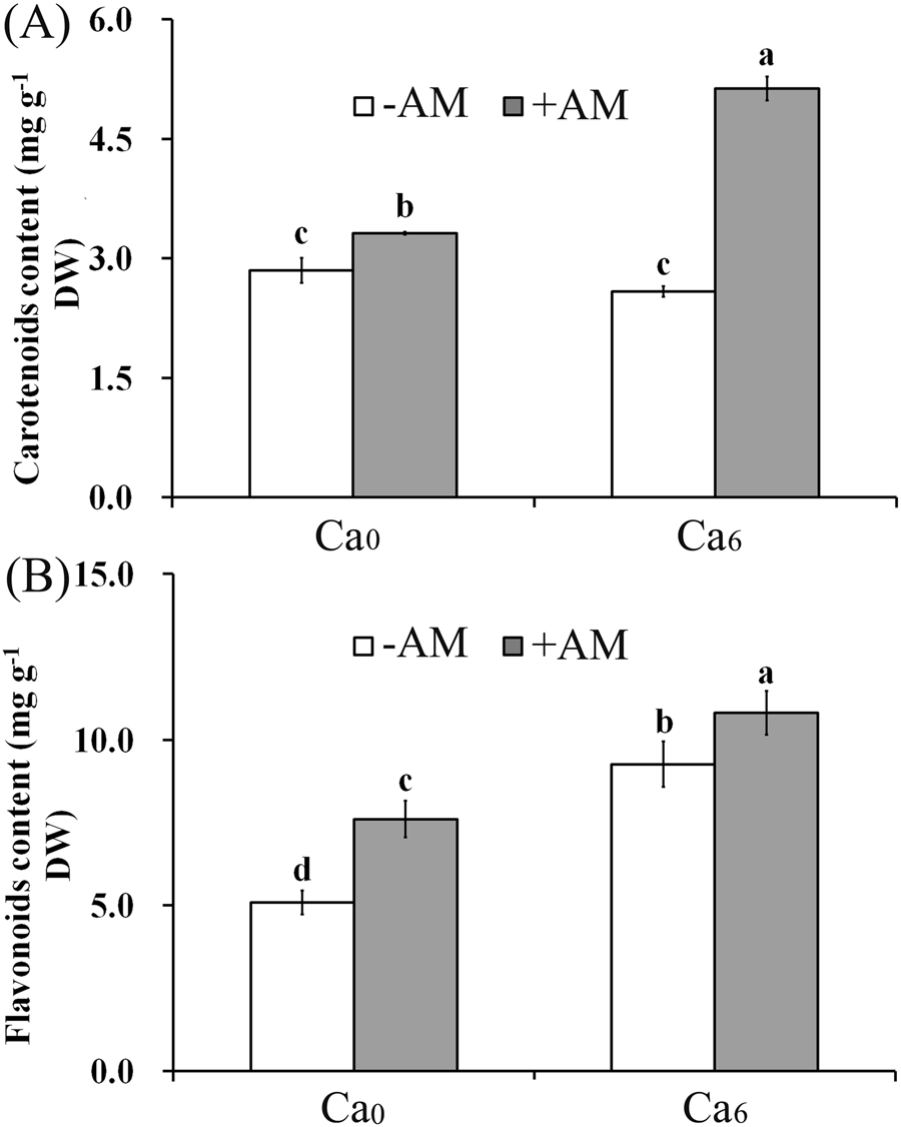
Quantification of carotenoids and flavonoids in roots of peanut seedlings. Carotenoids content (**A**) and total flavonoids content (**B**) were determined in roots of the AM plants and NM plants under Ca^2+^ deficient and sufficient conditions. Bars indicate means ± SD from six plants. Letters represent significant differences between treatments and the control (one-way ANOVA, *P* < 0.05). DW, dry weight.

Next, we verified whether transcriptional changes of carotenoid- and flavonoid-related genes impacted their respective content. As expected, total carotenoid content was higher in Ca_0_ + AM plants than the control and was the highest in Ca_6_ + AM plants, but was unchanged in Ca_6_ − AM plants (Fig. 5A). However, total flavonoid content was the highest in Ca_6_ treatments, and was higher than the control in Ca_0_ treatment (Fig. 5B); both increases were significant.

## Discussion

Calcium is an essential macronutrient for plant growth and development, and also plays various important roles as a secondary messenger. Prolonged Ca^2+^ deficiency limits root development^30^. In this study, AM symbiosis increased the Ca^2+^ content in peanut seedlings (Fig. 1D), because AMF increased the root surface and root projections, which promote plant uptake of nutrients^31^. Conversely, the increase in Ca^2+^ content enhanced potassium level in plants by enhancing the transcripts of genes encoding the potassium channel^32^, and together with AM symbiosis improved plant nutrient uptake^9^, thus increasing the shoot and root dry weight. This indicated that the interaction between AM symbiosis and exogenous Ca^2+^ benefited the growth of peanut seedlings.

Our previous study reported that AM symbiosis combined with exogenous Ca^2+^ was better than AM symbiosis or Ca^2^^+^ application alone at improving the growth of peanut seedlings^29^. This, together with our observations on plant dry weight, could explain why Ca^2+^ application strengthens the role of AM symbiosis in plant growth by further regulating a major overlap of transcriptional changes in roots of AM plants (380 out of 510 genes, approximately 74%). In addition, the establishment of AM symbiosis requires the expression of AM-specific marker genes^33^. In this study, Ca^2+^ further up-regulated and specifically induced the transcripts of AM-specific marker genes in AM plants. It is possible that the Ca^2+^-calmodulin association with CCaMK induces the phosphorylation of CYCLOPS and forms a complex in the presence of calcium, which acts in concert with GRAS  TFs such as DELLA proteins to initiate the expression of AM-specific marker genes that are necessary to establish the AM symbiosis^14^. These results suggest that Ca^2+^ plays a vital role in the formation of AM symbiosis.

GRAS family TF encoding DELLA protein is a positive regulator in the formation of AM associations^17,34^, and is also involved in GA biosynthesis as a negative regulator of GA signaling^35^. AM symbiosis up-regulation of GA-related genes and GA content in roots has been reported in *M*. *truncatula* and tomato^36,37^. Hence, the observed increase of GA_3_ content may be the factor involved in Ca^2+^ further up-regulating the transcripts of *DELLAs* and the genes encoding gibberellins 20-oxidase, which is a key enzyme that catalyzes the penultimate steps in GA biosynthesis. This result implied that AM symbiosis positively regulated the transcriptional changes involved in GA biosynthesis, and that Ca^2+^ strengthens this effect.

tZR is the major transport form of CKs from root to shoot in plants^22,38^. However, it has been reported that CKs act as a negative regulator in lateral root initiation, because overproduction of CKs inhibited lateral root initiation^39,40^. In this study, the lower tZR in roots of AM plants suggested that the genes encoding cytokinin dehydrogenase, which catalyze the irreversible degradation of cytokinin, were down-regulated by Ca^2+^ application. Reduced tZR content may be beneficial to the initiation of AM symbiotic roots. This result supports the finding in some studies that CKs might not be involved in the regulation of AM symbiosis development^16^. In addition, SA and carotenoid have been demonstrated to be activated by AM colonization^16,41^, and these activations were specific to AM symbiosis but not Ca^2+^ (Fig. 4), suggesting that increases in SA and carotenoid content can serve as AM-specific marker metabolites.

Flavonoid is involved in hyphal growth and branching^23^, and in turn, AMF benefit flavonoid biosynthesis and accumulation in roots of *M*. *truncatula*^42,43^. This is in line with our observation that more flavonoids were accumulated in roots of AM plants treated by Ca^2+^ application, which is attributed to Ca^2+^ inducing more transcripts of *DELLA* genes. DELLA-mediated signaling participates in regulating the accumulation of anthocyanin, one of the derivatives of flavonoids^44^. Additionally, the accumulation of SA can increase the flavonoid content in AM plants^45^. Thus, more flavonoids were observed in AM plants treated by Ca^2+^. These results suggested that Ca^2+^ and AM symbiosis might share the flavonoid biosynthetic pathway for improving plant growth.

**Figure 6:**
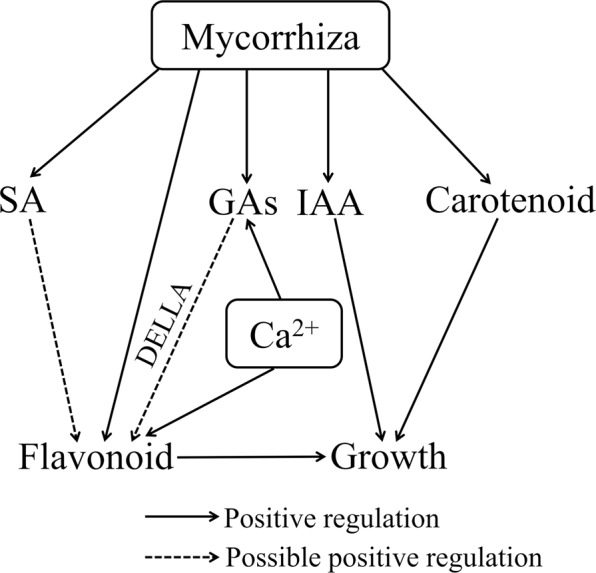
Proposed model of AM- and Ca^2+^- regulated pathways in peanut roots. AM symbiosis increases the content of IAA, GAs, SA, carotenoids, and flavonoids. Total flavonoids were also accumulated by regulating the transcripts of *DELLA* genes and the increase of SA in AM plants. Ca^2+^ application only increases the GA and flavonoid contents.

Based on our data, we propose a model of interactive pathways that modulate hormone levels, secondary metabolism, and ultimately the growth of AM and Ca^2+^ plants (Fig. 6). In this model, AM symbiosis promotes the growth of peanut seedlings by increasing contents of GAs, IAA, SA, carotenoids, and flavonoids. However, exogenous Ca^2+^ application only enhances the GA level and flavonoid content for improving plant growth. The increase in flavonoid content in AM symbiosis or Ca^2+^-treated plants may be a reason for the regulated* DELLA* that may enhance flavonoid accumulation. The proposed model reveals that synergy of AM symbiosis with Ca^2+^ promotes peanut growth by regulating GAs and flavonoid biosynthesis, but carotenoid and SA biosynthesis are specifically regulated by AM symbiosis. These findings should be validated in future research.

## Methods

### Plant material and growth conditions

Peanut cultivar ‘Huayu 22’ seeds were surface sterilized with 70% alcohol for 3 min and rinsed six times with sterile water. They were then germinated in the dark at 25 °C for 3 days. The germinated seeds were transferred to pots filled with quartz sand which was rinsed with deionized water 10 times to remove as much Ca^2+^ as possible, and then seeds were sterilized at 121 °C for 30 min. Half of the young seedlings were inoculated with about 300 *F*. *mosseae* spores (BEG HEB02); the other half were not colonized by *F*. *mosseae*. The peanut seedlings were grown in a greenhouse at 24 °C/18 °C with a 16/8 h photoperiod, at a photosynthetic photo flux density of 700 µmol·m^−2^·s^−1^, and 60% relative humidity. Each seedling was watered regularly with 80 ml of modified Hoagland’s solution (5 mM KNO_3_, 2 mM MgSO_4_·7H_2_O, 1 mM KH_2_PO_4_, 0.1 mM EDTA-Na_2_, 0.1 mM FeSO_4_·7H_2_O, 46 µM H_3_BO_4_, 0.32 µM CuSO_4_·5H_2_O, 0.77 µM ZnSO_4_·7H_2_O and 0.11 µM H_2_MoO_4_) supplemented with 6 mM Ca(NO_3_)_2_·4H_2_O (Ca^2+^ sufficient) or 6 mM NH_4_·NO_3_ (Ca^2+^ deficient, used for balancing nitrogen in Ca(NO_3_)_2_). There were four treatments: Ca_0_-AM, Ca_0_ + AM, Ca_6_ − AM, and Ca_6_ + AM, where 0 and 6 represent the Ca^2+^ concentrations (mM), + and − represent with or without inoculation of *F*. *mosseae* spores. In this study, 6 mM of Ca(NO_3_)_2_ was chosen according to our previous report^6^. Six weeks later, the shoots and roots of AM and NM plants were harvested and further analyzed.

### Mycorrhizal quantification and determination of dry weight and Ca^2+^ content

After six weeks, shoots and roots of the AM and NM plants under Ca^2+^-deficient or Ca^2+^-sufficient conditions were harvested separately. Young roots of AM plants were examined by light microscopy (OLYMPUS, CX41, Japan) to estimate the extent to which the roots had been colonized by hyphae and arbuscules^46^. The fresh shoots and roots were dried at 105 °C for 30 min, and then dried at 80 °C until a constant weight. The Ca^2+^ contents in the roots from the different treatments were determined according to Yang *et al*.^6^.

### RNA extraction and sequencing

Total RNA was isolated from roots of AM and NM plants, and then enrichment of mRNA and synthesis of cDNA were conducted^1^. The cDNA from three biological replicates composed of four plants in each treatment were sequenced using an Illumina HiSeq. 2000 Platform. After filtering, high quality clean reads were aligned with a reference genome (https://peanutbase.org/organism/Arachis/ipaensis) using HISAT^47^; on average 70.61% reads were mapped, indicating that the samples were comparable.

### RNA-Seq analysis and data deposition

After genome mapping, we used StringTie software to reconstruct transcripts with genome annotation information^47^, then identified novel transcripts using Cuffcompare and predicted the coding ability of those new transcripts using CPC software^48,49^. After novel transcript detection, the gene expression level was calculated for each sample with RSEM^50^. Based on the gene expression level, we used DEseq. 2 algorithms to detect differentially expressed genes (DEGs). A threshold of 1 for transcript ratio (log_2_FoldChange) in treatments versus control (Ca_0_-AM), and Padj (statistic of adjusted *P value*) ≤0.05 were set as criteria for the selection of DEGs in NM plants and AM plants under Ca^2+^-deficient and Ca^2+^-sufficient conditions. With DEGs, Gene Ontology (GO) classification and functional enrichment were performed using WEGO software^51^, and the pathway analyses were obtained using the KEGG database (https://www.genome.jp/kegg/pathway.html).

### Quantitative real-time PCR

To verify the RNA-Seq results, the expression levels of 15 selected genes were determined by quantitative RT-PCR. mRNA was isolated from the same samples sequenced by RNA-Seq, and the first-strand cDNAs were synthesized for qRT-PCR analyses using SYBR Premix Ex Taq polymerase (Takara) according to the manufacturer’s protocol. The designed primers are shown in Supplementary Table S4. The control reactions were conducted using primers Tua5-F and Tua5-R^52^. At least three replicates were tested per sample. Relative mRNA (fold) differences were assessed with the 2^− ΔΔCt^ formula^53^, the values were subsequently transformed to the log_2_ scale.

### Determination of plant hormones

The roots (fresh weight) were ground into a powder in liquid nitrogen, and 1.0 g of powder was used to determine the concentration of endogenous hormones by high performance liquid chromatography (HPLC)^54^, including IAA, tZR, and GA_3_. The SA content was measured according to a previous method^55^. Three independent replicates per sample were statistically analyzed.

### Carotenoid and flavonoid content analyses

Carotenoids were extracted from the roots of AM and NM plants^56^. Total carotenoid content in roots was calculated using absorbance at 450 nm. Flavonoids in roots were measured by chloride colorimetric assay^57^, and total flavonoid content was determined according to the standard curve of quercetin at an absorbance of 510 nm.

### Statistical analysis

Analysis of variance was performed using SSPS software version 16.0 for Windows. One-way analysis of variance (ANOVA) was used, followed by Duncan's test for multiple comparisons. The values obtained are the mean ± SE for the three replicates in each treatment. A *P* value ≤ 0.05 was considered to be significant.

## Supplementary information

Supplemental Figure S1

Supplemental Tables
