# Author Correction: Synergy of arbuscular mycorrhizal symbiosis and exogenous Ca^2+^ benefits peanut (*Arachis hypogaea* L.) growth through the shared hormone and flavonoid pathway

**DOI:** 10.1038/s41598-020-57448-2

**Published:** 2020-01-15

**Authors:** Li Cui, Feng Guo, Jialei Zhang, Sha Yang, JingJing Meng, Yun Geng, Xinguo Li, Shubo Wan

**Affiliations:** 1 Biotechnology Research Center, Shandong Academy of Agricultural Sciences, Jinan, 250100 China; 2 Scientific Observing and Experimental Station of Crop Cultivation in East China, Ministry of Agriculture, Jinan, 250100 China; 3 College of Life Sciences, Shandong Normal University, Jinan, 250014 China; 4 Shandong Academy of Agricultural Sciences and Key Laboratory of Crop Genetic Improvement and Ecological Physiology of Shandong Province, Jinan, 250100 China

Correction to: *Scientific Reports* 10.1038/s41598-019-52630-7, published online 07 November 2019

This Article contains errors.

The legend in Table 1 contains a typographical error where, “Differentially expressed genes of AM-specific markers in roots of Ca_0_ + AM and Ca_6_ + AM treated plants compared with controls. Values represent significant changes in roots of AM plants under Ca^2+^ deficient and sufficient conditions compared with the control (NM-Ca). Positive and negative ratios indicate up- and down-regulated genes. − Represents no significant alterations at log_2_FoldChange ≥ 1 and P value ≤ 0.05 level.”

should read:

“Differentially expressed genes of AM-specific markers in roots of Ca_0_ + AM and Ca_6_ + AM treated plants compared with controls. Values represent significant changes in roots of AM plants under Ca^2+^ deficient and sufficient conditions compared with the control. Positive and negative ratios indicate up- and down-regulated genes. − Represents no significant alterations at log_2_FoldChange ≥ 1 and P value ≤ 0.05 level.”

Table 2 contains a typographical error where,

“**GeneID Gene Description Ca0 + AM/CK Ca6 − AM/CK Ca6 + AM/CK”**

should Read:

“**GeneID Gene Description Ca**_**0**_ **+ AM/CK Ca**_**6**_ **− AM/CK Ca**_**6**_ **+ AM/CK”**

Additionally, the legend of Table 2 contains a typographical error where, “List of selected altered genes involved in hormone signal transduction in roots of Ca_0_+AM, Ca_6_—AM, and Ca_6_+AMtreated plants compared with controls. Values represent significant alterations in AM or Ca^2+^-treated plants compared with the control. Positive and negative ratios indicate up- and down-regulated genes. −Represents no significant alterations at log_2_FoldChange ≥ 1 and P ≤ 0.05 level.”

should Read:

“List of selected altered genes involved in hormone signal transduction in roots of Ca_0_+AM, Ca_6_—AM, and Ca_6_+AM treated plants compared with controls. Values represent significant alterations in AM or Ca^2+^-treated plants compared with the control. Positive and negative ratios indicate up- and down-regulated genes. −Represents no significant alterations at log_2_FoldChange ≥ 1 and P ≤ 0.05 level.”

Finally, this Article contains a typographical error in the Methods section under subheading ‘Plant material and growth conditions.’ where, “The germinated seeds were transferred to pots filled with quartz sand which was rinsed with deionized water 10 times to remove as much Ca^2+^ as possible, and then seeds were sterilized at 121 °C for 30 min.”

should read:

“The germinated seeds were transferred to pots filled with quartz sand which was rinsed with deionized water 10 times to remove as much Ca^2+^ as possible, and the quartz sand was sterilized at 121 °C for 30 min.”

