# Supplementary material for: Synergy of arbuscular mycorrhizal symbiosis and exogenous Ca2+ benefits peanut (Arachis hypogaea L.) growth through the shared hormone and flavonoid pathway
Source: Sci Rep. 2019 Nov 7;9:16281. doi: 10.1038/s41598-019-52630-7 (PMC6838158; doi:10.1038/s41598-019-52630-7)
Supplement: Supplementary file 1 — Supplemental Figure S1 [file 41598_2019_52630_MOESM1_ESM.docx]

**The synergy of arbuscular mycorrhizal symbiosis and Ca^2+^ benefits peanut (*Arachis hypogaea* L**.**) growth by sharing hormone and flavonoid pathway**

Li Cui^1,2†^, Feng Guo^1,2†^, Jialei Zhang^1,2^, Sha Yang^1,2^, JingJing Meng^1,2^, Yun Geng^1,2^, Xinguo. Li^1,2，3*^, Shubo Wan^2,4*^

1 Biotechnology Research Center, Shandong Academy of Agricultural Sciences, Jinan 250100, China

2 Scientific Observing and Experimental Station of Crop Cultivation in East China, Ministry of Agriculture, Jinan 250100, China

3 College of Life Sciences, Shandong Normal University, Jinan 250014, China

4 Shandong Academy of Agricultural Sciences and Key Laboratory of Crop Genetic Improvement and Ecological Physiology of Shandong Province, Jinan 250100, China

*Correspondence: xinguol@163.com; wanshubo2016@163.com

†These authors contributed equally to this work as co-first authors


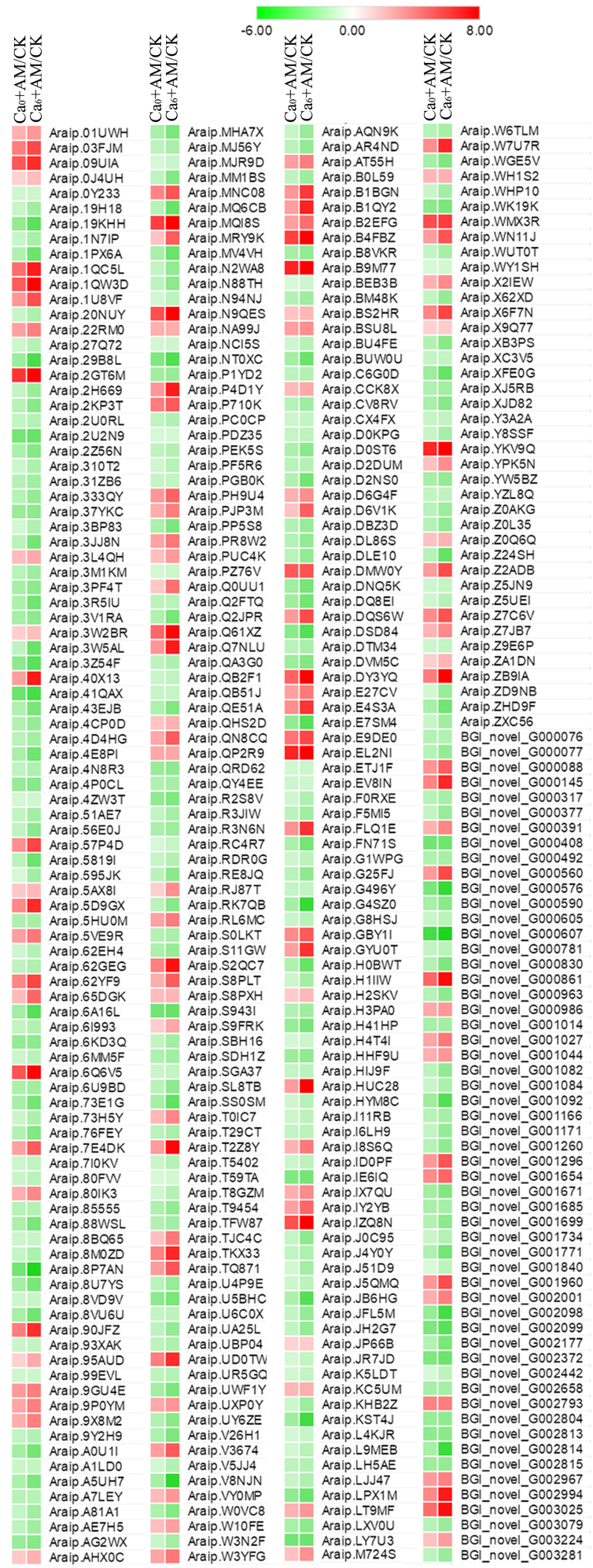


**Supplementary Figure S1**. List of co-regulated DEGs in roots of Ca_0_+AM plants and Ca_6_+AM plants. Red and green areas indicate up-regulated and down-regulated values, respectively.
