# Supplementary material for: Synergy of arbuscular mycorrhizal symbiosis and exogenous Ca2+ benefits peanut (Arachis hypogaea L.) growth through the shared hormone and flavonoid pathway
Source: Sci Rep. 2019 Nov 7;9:16281. doi: 10.1038/s41598-019-52630-7 (PMC6838158; doi:10.1038/s41598-019-52630-7)
Supplement: Supplementary file 2 — Supplemental Tables [file 41598_2019_52630_MOESM2_ESM.docx]

**The synergy of arbuscular mycorrhizal symbiosis and Ca^2+^ benefits peanut (*Arachis hypogaea* L**.**) growth by sharing hormone and flavonoid pathway**

Li Cui^1,2†^, Feng Guo^1,2†^, Jialei Zhang^1,2^, Sha Yang^1,2^, JingJing Meng^1,2^, Yun Geng^1,2^, Xinguo. Li^1,2，3*^, Shubo Wan^2,4*^

1 Biotechnology Research Center, Shandong Academy of Agricultural Sciences, Jinan 250100, China

2 Scientific Observing and Experimental Station of Crop Cultivation in East China, Ministry of Agriculture, Jinan 250100, China

3 College of Life Sciences, Shandong Normal University, Jinan 250014, China

4 Shandong Academy of Agricultural Sciences and Key Laboratory of Crop Genetic Improvement and Ecological Physiology of Shandong Province, Jinan 250100, China

*Correspondence: xinguol@163.com; wanshubo2016@163.com

†These authors contributed equally to this work as co-first authors

**Supplementary Table S1.** List of conversely regulated DEGs in the roots of Ca_0_+AM plants and Ca_6_+AM plants.

| **Gene ID** | **Description** | **Ca_0_+AM/CK** | **Ca_6_+AM/CK** |
| --- | --- | --- | --- |
| BGI_novel_G000157 | unknown protein | 2.56 | 2.54 |
| Araip.QA79V | laccase/diphenol oxidase family protein | 1.66 | 1.64 |
| BGI_novel_G002038 | hypothetical protein | -1.93 | -1.90 |
| Araip.Q85BX | unknown protein | -4.94 | -4.86 |
| Araip.H3ANA | 4-hydroxyphenylpyruvate dioxygenase | -1.38 | -1.35 |
| Araip.WR1Z1 | hypothetical protein | 5.33 | 5.22 |
| Araip.3P203 | Putative zinc finger protein CONSTANS-LIKE 11 | -2.05 | -2.01 |
| Araip.I0HKJ | Putative xyloglucan endotransglucosylase/hydrolase protein 32 | 1.82 | 1.78 |
| Araip.VAX9L | ACT-domain-containing protein kinase | -1.75 | -1.71 |
| Araip.5Z7DV | high mobility group B protein 15-like | -2.52 | -2.44 |
| Araip.RXZ9L | glycoside hydrolase family 1 protein | -1.44 | -1.39 |
| Araip.HRR0E | hypothetical protein VIGAN_10151800 | -2.69 | -2.59 |
| BGI_novel_G000510 | 2,3-bisphosphoglycerate-independent phosphoglycerate mutase | 2.12 | 2.04 |
| BGI_novel_G003140 | protein DETOXIFICATION 49-like | -2.04 | -1.95 |
| Araip.49GZ0 | Cytochrome P450 78A4 | 2.57 | 2.45 |
| Araip.UNK6B | uncharacterized protein LOC100789333 | 2.17 | 2.06 |
| BGI_novel_G002191 | probable acyl-activating enzyme 5, peroxisomal | -1.19 | -1.12 |
| Araip.E7CF7 | bifunctional 3-dehydroquinate dehydratase | -1.44 | -1.35 |
| Araip.XI1X3 | nodulin-like/MFS transporter | -3.34 | -3.12 |
| BGI_novel_G000606 | putative disease resistance protein At3g14460 | -1.51 | -1.40 |
| Araip.509FX | hypothetical protein POPTR_0008s02350g | 1.31 | 1.22 |
| Araip.VF8KB | probable tyrosine-protein phosphatase | -1.21 | -1.11 |
| Araip.SCU5A | hypothetical protein glysoja_011334 | -2.04 | -1.87 |
| Araip.MA9YP | Transcription factor bHLH25 | -2.52 | -2.30 |
| Araip.31HZX | protein IQ-DOMAIN 1-like | 1.13 | 1.03 |
| Araip.EMI9Y | cytochrome P450 81E8 | -1.16 | -1.04 |
| Araip.4A201 | hypothetical protein glysoja_028637 | 4.58 | 4.11 |
| Araip.XZD1G | uncharacterized protein LOC100305614 | -1.64 | -1.47 |
| BGI_novel_G001039 | unknown protein | 2.83 | 2.52 |
| Araip.X98AS | tubby C 2 protein [Medicago truncatula] | -2.53 | -2.21 |
| Araip.V3M2B | hypothetical protein LR48_Vigan03g254500 | 5.35 | 4.67 |
| Araip.T1IGL | Cytochrome P450 82A4 | -2.59 | -2.23 |
| Araip.AKH01 | Basic blue protein | 1.87 | 1.58 |
| Araip.R261D | serine carboxypeptidase II-3-like | 5.44 | 4.32 |
| Araip.KN8Z5 | IQ domain-containing protein IQM1-like | -4.07 | -3.22 |
| Araip.TA0RC | uncharacterized protein LOC105649658 | 4.44 | 3.49 |
| Araip.4E5HU | hypothetical protein PHAVU_003G287800g | -2.28 | -1.77 |
| Araip.NB6VC | hypothetical protein GLYMA_11G170300 | -1.62 | -1.23 |
| Araip.UT2GI | uncharacterized protein LOC101507941 | -2.60 | -1.87 |
| Araip.7IS7K | thiosulfate sulfurtransferase 18-like | 2.05 | 1.29 |

Values represent significant alterations in AM or Ca^2+^ plants versus the control (transcript ratio of at least 1, Paj < 0.05). Positive and negative ratios indicate up- and down-regulated genes. - represents no significant alterations at log2FoldChange >1 and Padj < 0.05 level.

**Supplementary Table S2.** List of DEGs involved in Ca and Ca^2+^ signal pathway in roots of Ca_0_+AM, Ca_6_-AM and Ca_6_+AM plants compared with the control.

| **Gene ID** | **Gene Description** | **Ca_0_+AM/CK** | **Ca_6_-AM/CK** | **Ca_6_+AM/CK** |
| --- | --- | --- | --- | --- |
| Araip.1C4CW | hypothetical protein VIGAN_10177100 | − | − | -1.98 |
| Araip.1Z5U3 | potassium channel KAT3 protein | − | − | 3.71 |
| Araip.7HI2H | tonoplast intrinsic protein | − | − | 1.50 |
| Araip.FP1A1 | uncharacterized protein LOC100306092 isoform X1 | − | − | 1.39 |
| Araip.G4LHX | putative calcium-transporting ATPase 13, plasma membrane-type | − | − | -1.23 |
| Araip.H76LJ | hypothetical protein LR48_Vigan07g200900 | − | − | -1.59 |
| Araip.HLU05 | probable NAD(P)H dehydrogenase (quinone) FQR1-like 2 | − | − | 1.04 |
| Araip.LE8QH | probable NAD(P)H dehydrogenase (quinone) FQR1-like 2 | − | − | 1.22 |
| Araip.LLP3C | cyclic nucleotide-gated ion channel 4-like isoform X1 | − | − | 1.80 |
| Araip.PCB4M | calcium-transporting ATPase 9, plasma membrane-type-like | − | − | 1.47 |
| BGI_novel_G002490 | nuclear transcription factor Y subunit C-1-like | − | − | 3.75 |
| Araip.R6YEY | Potassium channel AKT2/3 | − | − | 2.38 |
| Araip.SD8AP | MLO protein like 1 | − | − | 2.15 |
| Araip.TWW4R | cold-induced wall associated kinase | − | − | -2.19 |
| Araip.UB1TB | aquaporin TIP1-2-like | − | − | 4.11 |
| Araip.WA456 | annexin | − | − | 3.08 |
| Araip.XJU6V | probable aquaporin TIP2-2 | − | − | 1.25 |
| BGI_novel_G002793 | probable NAD(P)H dehydrogenase (quinone) FQR1-like 2 | 2.93 | − | 3.07 |
| Araip.JFL5M | respiratory burst oxidase homolog protein B-like | -1.56 | -2.34 | -2.77 |
| Araip.Z0Q6Q | annexin-like protein | 1.47 | 1.43 | 1.79 |
| BGI_novel_G001260 | calcium-binding protein CAST | -1.90 | -2.23 | -2.90 |
| Araip.PR8W2 | Flavoprotein wrbA | 2.18 | 2.29 | 3.22 |
| Araip.QRD62 | calcium uptake protein 1, mitochondrial-like | -2.65 | -3.64 | -2.95 |
| Araip.1W80J | kinesin-like protein NACK2 | − | 1.99 | 1.99 |
| Araip.2U42B | vacuolar cation/proton exchanger 3 isoform X6 | − | 4.23 | 2.51 |
| Araip.54USS | calmodulin-binding family protein | − | -1.98 | -2.37 |
| Araip.87TAW | Calcium-responsive transactivator | − | 1.41 | 1.31 |
| Araip.H5FJR | wall-associated receptor kinase-like 1 isoform X2 | − | -1.93 | -2.12 |
| Araip.I0SI9 | Vacuolar cation/proton exchanger 1 | − | 3.71 | 4.21 |
| Araip.L73J1 | Potassium channel SKOR | − | -1.49 | -1.72 |
| Araip.WIW54 | wall-associated receptor kinase-like 14 | − | -2.00 | -2.21 |
| Araip.UY1E7 | calmodulin-binding transcription activator 4-like isoform X1 | − | -1.36 | -1.57 |
| Araip.5FF6E | Concanavalin A-like lectin/glucanase | − | -1.36 | − |
| Araip.7E4FS | kinesin-like protein FRA1 isoform X3 | − | 1.97 | − |
| Araip.95KUY | patatin-like protein 6 | − | -1.23 | − |
| Araip.F3SVI | calmodulin-domain kinase CDPK protein | − | -2.55 | − |
| Araip.J58EQ | Annexin-like protein RJ4 | − | 2.48 | − |
| Araip.Q94B9 | kinesin-like protein KIN12A | − | 1.12 | − |
| Araip.S8WH0 | plant calmodulin-binding-like protein | − | -1.02 | − |
| Araip.SED3F | putative cyclic nucleotide-gated ion channel 15 | − | -2.19 | − |
| Araip.TRR5D | cysteine-rich receptor-like protein kinase 10 isoform X1 | − | -2.82 | − |
| Araip.UHZ8X | calcium-dependent protein kinase 28-like | − | -1.00 | − |
| Araip.URB55 | respiratory burst oxidase homolog protein B | − | -1.61 | − |
| Araip.WA9TW | wall-associated receptor kinase-like protein | − | 1.50 | − |
| Araip.WW1MI | cysteine-rich receptor-like protein kinase 3 | − | -1.10 | − |
| Araip.Z8ALS | MLO-like protein 4 | − | 1.48 | − |
| BGI_novel_G003112 | sarcoplasmic reticulum histidine-rich calcium-binding protein-like | − | -1.18 | − |

Values represent significant alterations in AM or Ca^2+^ plants versus the control (transcript ratio of at least 1, Paj < 0.05). Positive and negative ratios indicate up- and down-regulated genes. - represents no significant alterations at log2FoldChange >1 and Padj < 0.05 level.

**Supplementary Table S3**. Differently expressed genes involved in flavonoids biosysthesis in roots of AMF and Ca^2+^ treated plants compared with the control.

| **Gene ID** | **Gene Description** | **Ca_0_+AM/CK** | **Ca_6_-AM/CK** | **Ca_6_+AM/CK** |
| --- | --- | --- | --- | --- |
| Araip.5S2PS | chalcone synthase | − | -2.02 | -1.85 |
| Araip.Z5UEI | chalcone synthase | -1.23 | -1.50 | -1.94 |
| Araip.THJ4F | chalcone synthase | − | -1.66 | -1.83 |
| Araip.YF2QF | chalcone synthase | − | − | -1.36 |
| Araip.E9KQ7 | chalcone synthase | − | − | -2.23 |
| Araip.U7CA5 | chalcone synthase | − | − | -1.29 |
| Araip.BU4FE | chalcone synthase | -1.91 | -1.52 | -1.91 |
| Araip.62EH4 | chalcone synthase | -1.27 | -1.64 | -1.97 |
| Araip.TTM09 | chalcone synthase | − | − | -1.19 |
| BGI_novel_G001084 | chalcone synthase | -1.42 | -1.53 | -2.21 |
| Araip.PF5R6 | chalcone synthase | -1.26 | -1.91 | -1.91 |
| Araip.3T4SK | chalcone synthase | − | − | -1.32 |
| Araip.QL51L | chalcone synthase | − | -1.23 | -1.71 |
| Araip.V9QDS | chalcone synthase | − | -1.35 | -1.70 |
| Araip.E7BUX | chalcone synthase | − | − | -1.18 |
| BGI_novel_G001082 | chalcone synthase | -1.72 | -1.97 | -2.85 |
| Araip.LS9BW | chalcone synthase | − | -1.37 | -1.72 |
| Araip.YDG4K | chalcone synthase | − | − | -1.49 |
| Araip.Q8ZS3 | chalcone synthase | − | -1.73 | -1.75 |
| Araip.S9JRR | chalcone synthase | − | − | -1.40 |
| BGI_novel_G001083 | chalcone synthase | − | − | -1.48 |
| Araip.8ZH5X | chalcone synthase | − | -1.20 | -1.64 |
| Araip.73H5Y | chalcone synthase | -1.35 | -1.47 | -2.11 |
| Araip.QB1YM | chalcone synthase | − | -1.18 | − |
| Araip.V284D | chalcone isomerase | − | -1.04 | -1.00 |
| Araip.6PA6C | flavonol synthase | − | − | 1.87 |
| Araip.NI1B2 | flavonol synthase | − | -1.65 | -1.69 |
| Araip.MJ56Y | flavonol synthase | -2.22 | -2.02 | -2.22 |
| Araip.BEB3B | flavonol synthase | -1.31 | -1.35 | -1.31 |
| Araip.68F0J | flavonol synthase | − | − | -1.54 |
| Araip.MWP7Z | flavonol synthase | − | -2.76 | -2.17 |
| Araip.FWS96 | flavonol synthase | − | -1.60 | -2.08 |
| Araip.Q25ER | trans-cinnamate 4-monooxygenase | − | -2.53 | -2.63 |
| Araip.56XNF | leucoanthocyanidin dioxygenase | − | -2.31 | -2.84 |
| Araip.E99A6 | leucoanthocyanidin reductase | − | -1.26 | -1.30 |
| Araip.XGB85 | caffeoyl-CoA O-methyltransferase | − | -3.44 | -2.82 |
| Araip.JD4SK | caffeoyl-CoA O-methyltransferase | − | 1.76 | − |
| Araip.Z2KAD | shikimate O-hydroxycinnamoyltransferase | − | − | 2.70 |
| BGI_novel_G001027 | shikimate O-hydroxycinnamoyltransferase | 2.09 | 2.18 | 3.18 |
| Araip.8C6BV | bifunctional dihydroflavonol 4-reductase/flavanone 4-reductase | − | 1.40 | 1.22 |
| Araip.LA8CL | Polyketide reductase | − | -1.18 | − |
| BGI_novel_G002117 | Polyketide reductase | − | -1.03 | − |

Values represent significant alterations in AM or Ca^2+^ plants versus control (transcript ratio of at least 1, Paj < 0.05). Positive and negative ratios indicate up- and down-regulated genes. - represents no significant alterations at log2FoldChange >1 and Padj < 0.05 level.

**Supplementary** **Table S4**. List of primers used for quantitative RT-PCR.

| **Gene Name** | **Gene ID** | **Forward Primer (5'-3')** | **Reverse Primer (5'-3')** |
| --- | --- | --- | --- |
| WRKY transcription factor | Araip.LH5AE | CCGCCTCCATGCTCCTCTCC | GCTGAAGCTGAGAGTGTAGCCATG |
| WRKY transcription factor 2 | Araip.9P0YM | GGTCGTAGGAAGGCAATTGAGGAG | TTCACCATCAACACCACCATCACC |
| very-long-chain 3-oxoacyl-CoA reductase | Araip.4N8R3 | FCCGCCATCACCGCCAAGTTC | TCCTCATGCCGTCGTCGAGATC |
| adrenodoxin-NADP+ reductase | BGI_novel_G000772 | CCGTCGCCTTCAGCAGTGTTC | AGGTAGAATCAGGACCGCAGGAC |
| auxin-responsive protein IAA | Araip.I2M0Y | TCTGATCCTGCCAAGCCTCCTG | TGCACCATCCATGCTTACCTTCAC |
| chalcone synthase | Araip.Z5UEI | CGGTGTTGCGTTGCCTGGAG | GCGAAGCAGCCTTGGTGGTAC |
| pectinesterase | Araip.8P7AN | CCTTCCGTTCCGCCACTGTTG | TTGCTGCACCTGCTGTGTTCC |
| leucine-rich repeat protein SHOC2 | BGI_novel_G000607 | AGTGTATTGTGGTGTCACGCTCTC | GCTGAGGTGCTGCCATCCATAG |
| UDP-glucosyl transferase | Araip.Y8SSF | CCATCCACCTCATCATGCTCACG | CGCTAAGAGATCGCCGAACCAC |
| transcription factor VIP1 | Araip.LXV0U | GGATGGATGAGTTGACGGCGAAG | CCACAGAAGACGACGACGAACAC |
| pyridoxal phosphate phosphatase PHOSPHO2 | Araip.W3YFG | CTTCCCACCACGCTTTGGAA | GCTGCCTCAATAGCATGCAC |
| carlactone synthase | Araip.MNC08 | CACCGCCAACTGGAGTCACAAG | CGGAGAACAGGCTCGCCAATTC |
| serine carboxypeptidase-like clade II | Araip.R261D | ATGCACTCAACGTCCTCAAGCTC | GCACCGCATATCCTCCAATCTCG |
| flavonol synthase | Araip.MJ56Y | GCCATGGATGACATGCTCCTAGC | CCGGTGGTTGCAAGTAAGATGAGG |
| protein transport protein SEC23 | Araip.GBY1I | TTCGTGTCACTGGCTCTATTCGTG | TGGCACAAGGCACATGGATCAC |
